# Complete Septate Uterus With Cervical Duplication and Vaginal Septum (U2b C2 V1): A Rare Müllerian Malformation With Diagnostic and Management Challenges

**DOI:** 10.7759/cureus.88794

**Published:** 2025-07-26

**Authors:** Khadija Elaitari, Soukaina Jabour, Nadia Boujida, Nazik Allali, Latifa Chat, Nada Douraidi, Salma Tahri Jautei, Rim Abboudi, Fatima El Hassouni, Samir Bargach, Siham El Haddad

**Affiliations:** 1 Department of Radiology, Rabat Children's Hospital, Rabat, MAR; 2 Department of Obstetrics and Gynecology, Oncology and High-Risk Pregnancies, Souissi Maternity Hospital, Rabat, MAR

**Keywords:** classification, embryology, management, mri, müllerian anomaly, u2bc2v1, ultrasound

## Abstract

Complex Müllerian anomalies, such as a complete septate uterus (U2b), cervical duplication (C2), and a non-obstructive longitudinal vaginal septum (V1), are rare malformations with intricate embryological origins. They often remain undiagnosed in the absence of specific clinical symptoms. This case highlights the diagnostic value of pelvic magnetic resonance imaging (MRI) and the therapeutic benefit of personalized surgical management. In our patient, surgical resection of the vaginal septum led to significant symptomatic improvement, including the restoration of a satisfactory sexual life.

## Introduction

Congenital uterine anomalies (CUAs) are malformations of the female reproductive tract caused by abnormalities during the fusion or resorption processes of the Müllerian ducts. These anomalies include septate, bicornuate, unicornuate, and didelphys uteri and are commonly classified using systems such as those of the American Society for Reproductive Medicine (ASRM) or the European Society of Human Reproduction and Embryology (ESHRE/ESGE). They are estimated to affect about 5.5% of women in the general population [1].

We report the case of a woman presenting with a rare and complex Müllerian anomaly, diagnosed late at the age of 46 following a non-penetrative sexual intercourse. Magnetic resonance imaging (MRI) and 3D ultrasound are the most commonly used modalities for establishing this type of diagnosis [2]. Surgical treatment is not systematically indicated due to the absence of standardized therapeutic protocols for this type of malformation [1,3]. In the present case, resection of the vaginal septum was performed to allow the patient to achieve a satisfactory sexual life.

## Case presentation

**Figure 1:**
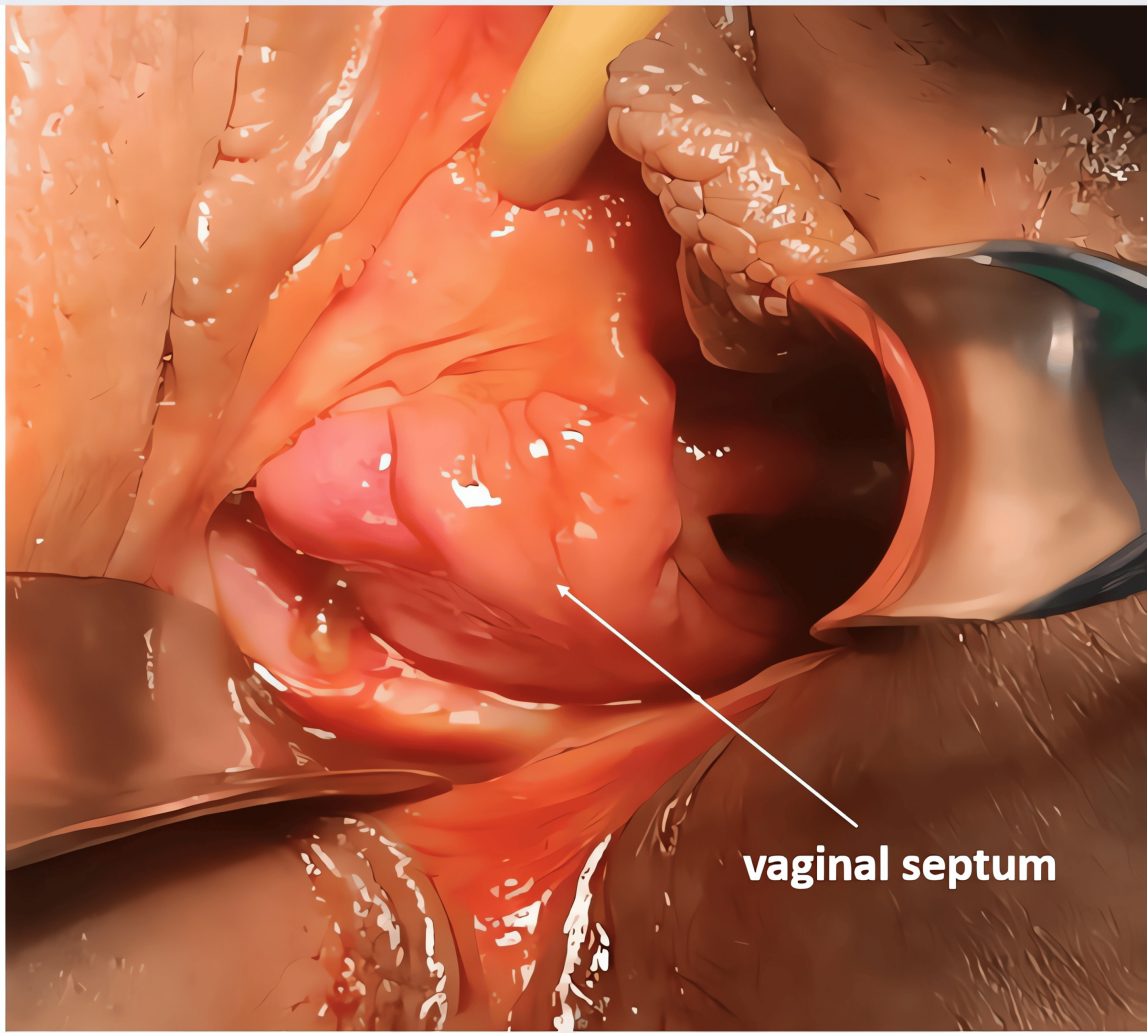
Image showing a broad longitudinal vaginal septum

A 46-year-old woman with no significant medical history was admitted to the gynecological emergency department for dyspareunia accompanied by mild bleeding following her first sexual intercourse with her husband. On examination, the patient was hemodynamically stable. The gynecological examination was difficult to perform and required anesthesia to allow for a more thorough exploration. This revealed a thick, non-communicating longitudinal vaginal septum and two cervical orifices (Figure 1). A small right lateral vaginal tear was also noted. Until then, the patient had reported no dyspareunia, dysmenorrhea, or chronic pelvic pain.

**Figure 2:**
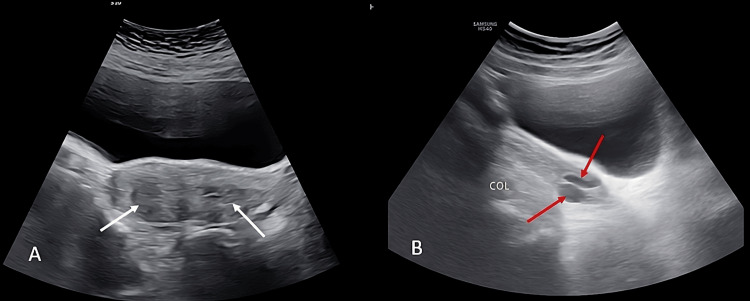
Suprapubic ultrasound showing two non-divergent uterine cavities (A: white arrow) and two cervical canals (B: red arrow)

Given the suspicion of a Müllerian anomaly with a longitudinal vaginal septum, a 2D suprapubic pelvic ultrasound was performed as the first-line imaging modality. It revealed two non-divergent uterine cavities and two cervical canals (Figure 2). Transvaginal imaging could not be performed due to the altered vaginal anatomy. The absence of 3D imaging technology did not allow for a mid-coronal uterine view, thereby limiting the evaluation of the external uterine contour as well as internal and external indentations.

**Figure 3:**
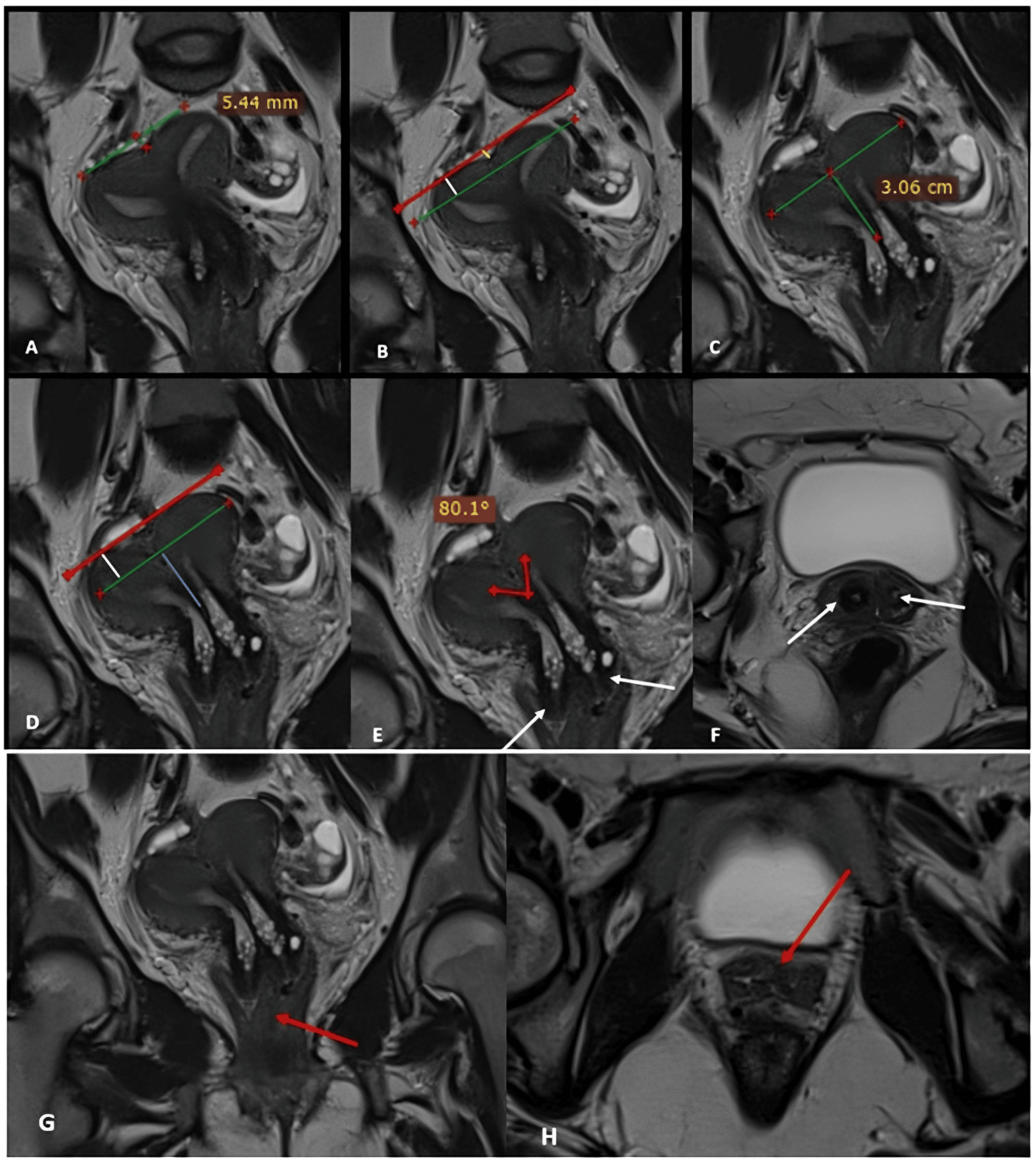
Pelvic MRI in T2-weighted coronal and axial sections showing a septate uterus (A-E), duplicated cervix (E, F; white arrow), and longitudinal vaginal septum (G, H; red arrow) MRI: magnetic resonance imaging

An MRI of the pelvis was subsequently performed. It demonstrated a uterine malformation characterized by an external fundal indentation measuring 5.44 mm (<10 mm) (Figure 3A), or <50% of the myometrial thickness (Figure 3B); and an internal indentation measuring 30 mm (>10 mm) (Figure 3C), or >50% of the uterine wall thickness (Figure 3D), with an internal indentation angle measured at 80° (Figure 3E). The indentation extended to the internal os, fulfilling the diagnostic criteria for a septate uterus (U2b) according to the ASRM 2021 and ESHRE/ESGE 2013 classifications. 

This anomaly was associated with a duplicated cervix partially fused at the proximal portion, presenting two distinct external cervical os (Figure 3E and Figure 3F), and a non-obstructive longitudinal vaginal septum (Figure 3G and Figure 3H). These findings are consistent with a complete septate uterus with duplicated cervices and a longitudinal vaginal septum, classified as U2b C2 V1 according to the ASRM 2021 and ESHRE/ESGE 2013 systems, respectively.

**Figure 4:**
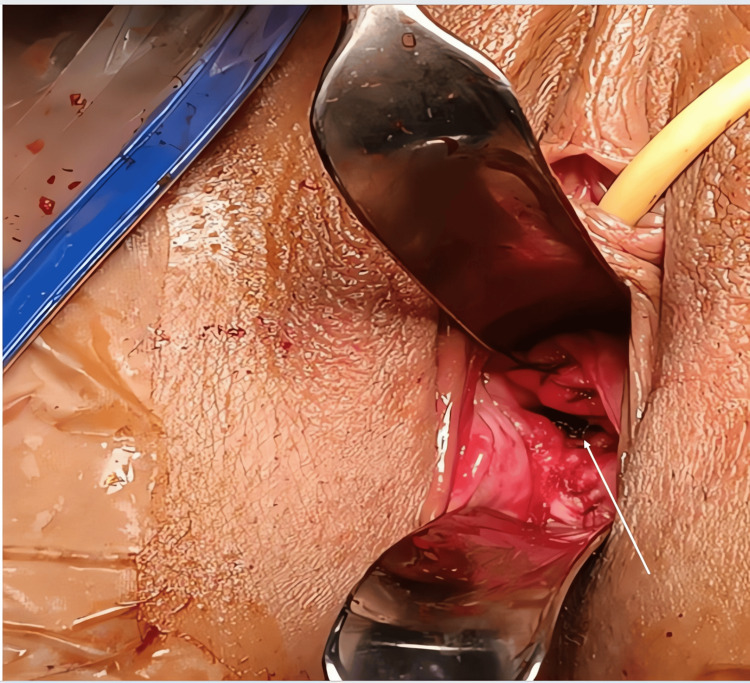
Postoperative image showing a single vaginal canal (arrow) following resection of the longitudinal vaginal septum, with no residual septal tissue visible

Once the diagnosis of a complete septate uterus associated with cervical duplication and a non-obstructive longitudinal vaginal septum (classified as U2b C2 V1 according to ESHRE/ESGE) was confirmed, a discussion was held with the patient and her husband. They consented to surgical intervention for resection of the vaginal septum to allow for normal sexual intercourse. The surgery was performed under spinal anesthesia, with the patient positioned in the gynecological position. After careful exploration of the malformation, the longitudinal vaginal septum was incised and excised using a cold knife technique. Hemostasis was achieved by placing sutures on the anterior and posterior vaginal walls (Figure 4). The remainder of the procedure was uneventful. The patient was reviewed three weeks postoperatively, and the outcome was excellent. She was able to resume sexual activity without dyspareunia or other symptoms.

## Discussion

Epidemiological profile

CUAs refer to developmental defects of the female reproductive tract, arising from incomplete or faulty fusion and resorption of the Müllerian ducts during embryogenesis. Their occurrence is estimated at around 5.5% in the general population, increasing to approximately 8% in infertile patients and up to 13.3% in women with recurrent pregnancy loss [1].

In recent years, the reported incidence of CUAs has increased, likely due to the widespread use of transvaginal ultrasound and MRI imaging, which allow for improved detection [1].

The most common form of CUAs is the isolated septate uterus, accounting for approximately 35% of all CUAs, with an estimated prevalence of 1% to 2% in the general population [3]. A rare variant of Müllerian development involves a septate uterus associated with cervical duplication and a non-obstructive longitudinal vaginal septum. This specific configuration was first described by McBean and Brumsted and is classified as U2b C2 V1 according to the 2013 ESHRE/ESGE classification [4]. Despite advances in imaging and classification systems, the true incidence of this triad remains unknown [3].

Embryological basis of Müllerian development

During female fetal development, the mesonephric ducts undergo regression due to the lack of testosterone. In contrast, the paramesonephric (Müllerian) ducts persist and differentiate under the influence of maternal and placental hormones, in the absence of anti-Müllerian hormone. These ducts give rise to the uterus, fallopian tubes, cervix, and the upper portion of the vagina, typically encompassing the upper two-thirds [2].

Müllerian anomalies are typically categorized into three main groups: those related to underdevelopment (such as hypoplastic or unicornuate uterus), those involving incomplete fusion of the Müllerian ducts (including didelphys and bicornuate uterus), and those resulting from failure of resorption (notably septate uterus). Vaginal septa, whether longitudinal or transverse, are thought to stem from disruptions in the lateral or vertical fusion processes, respectively [2].

Early research on the embryological development of the female reproductive system proposed that the uterus results from the merging of the two Müllerian ducts, followed by a one-way process of septal resorption that progresses from the lower (caudal) to the upper (cephalic) end [3,4]. Nevertheless, several case reports have documented Müllerian anomalies that do not fit this classification, indicating variability in the extent of fusion at the upper portion and resorption at the lower portion during embryogenesis [2].

The U2b C2 V1 type malformation cannot be explained by the traditional embryological theory, thereby challenging the classical model of Müllerian development [3,4]. This type of anomaly aligns more closely with an alternative approach proposed by Musses in 1967, in which the uterus, cervix, and upper portion of the vagina develop in three successive stages: fusion of the mid-segments of the Müllerian ducts begins centrally, then progresses simultaneously toward the caudal and cephalic ends [4]. In addition, the theory of bidirectional resorption postulates that septal resorption begins at the uterine isthmus, with concomitant progression toward the cranial and caudal poles. This concept was originally introduced by McBean et al. in 1994 [3].

Clinical manifestations

CUAs have been linked to a broad spectrum of reproductive outcomes, ranging from uneventful full-term pregnancies to complications such as miscarriage, premature delivery, abnormal fetal positioning, and a higher likelihood of cesarean delivery [1,5].

Among these anomalies, the uterine septum is particularly associated with significant reproductive implications, including infertility, repeated miscarriages, and various obstetric complications such as early delivery or restricted fetal development. The literature suggests that an abnormal vascular supply to the septum may lead to infertility through impaired implantation or spontaneous miscarriages, supporting the notion that surgical correction to restore a normal uterine cavity may represent the most effective therapeutic approach. However, as infertility is a multifactorial condition, it remains difficult to attribute causality solely to a septate uterus. This complexity is further supported by the existence of cases in which women with a septate uterus have had normal pregnancies without a history of infertility [1,3].

In rare situations, a uterine septum may be found in conjunction with a duplicated cervix and a longitudinal vaginal septum. When non-obstructive, the latter is commonly reported in association with dyspareunia [3]. In pediatric or adolescent patients, an obstructive longitudinal vaginal septum can present with symptoms of menstrual outflow obstruction from the time of menarche [2].

Diagnostic strategy and morphological classifications

Thanks to advances in modern imaging techniques, particularly 3D ultrasound and MRI, the diagnosis of uterine malformations can now be established without resorting to invasive techniques such as laparoscopy or hysteroscopy [1,6].

Ultrasound examinations using 2D transabdominal and endovaginal approaches continue to be the primary imaging techniques for assessing the female reproductive organs, especially when Müllerian duct anomalies involve the uterus, cervix, or vagina. A 3D ultrasound and MRI provide precise visualization of the external contour of the uterine fundus as well as the internal indentation of the endometrial cavity, particularly well appreciated on the coronal plane. These two morphological criteria are fundamental for making a reliable diagnosis of CUAs [2].

There are several classification systems for Müllerian developmental anomalies, each based on specific criteria, particularly for the diagnosis of a septate uterus. The most widely used systems in the literature are those of the ASRM and the ESHRE/ESGE [2].

The ASRM and ESHRE/ESGE classification systems differ significantly in their definitions of a septate uterus. Both employ a horizontal reference line, known as the interstitial or interostial line, drawn in the median coronal plane of the uterus, connecting the uterine ostia of the Fallopian tubes. The external indentation corresponds to the distance between a line drawn through the apexes of the right and left uterine horns and the deepest point of the uterine fundal indentation. The internal indentation is defined as the depth between the interstitial line and the lowest point of the indentation of the endometrial cavity [2].

Based on the 2016 ASRM guidelines, a septate uterus is characterized by a convex or flat external contour or an external indentation <1 cm, an internal indentation >1.5 cm, and an internal indentation angle <90°. The criteria were revised in the updated 2021 ASRM guidelines, which redefine a septate uterus as having an internal indentation >1 cm, with an internal indentation angle <90° [2,7].

In the ESHRE/ESGE classification system, the criteria for external and internal indentations are defined based on the thickness of the uterine wall, measured on an image in the median coronal plane between the interostial line and the external uterine contour. According to these criteria, a septate uterus (classified U2) is defined by a straight external contour or an external indentation <50%, associated with an internal indentation exceeding 50% of the uterine wall thickness. A partially septate uterus (classified U2a) presents an incomplete septum that stops above the internal cervical os, whereas a completely septate uterus (classified U2b) is characterized by a septum extending to the internal cervical os [2,8].

A double cervix, classified C2 in the ESHRE/ESGE classification, corresponds to a cervical fusion defect. It is characterized by the presence of two distinct cervical canals with two rounded external orifices (external os), which may be completely separated or partially fused. This configuration allows it to be distinguished from a septate cervix, which presents a single external os [2,8].

The longitudinal vaginal septum results from a lateral fusion defect and is classified V1 in the ESHRE/ESGE classification when non-obstructive. On T2-weighted MRI, it appears as a hypointense band extending along the length of the vagina, whose length is best visualized on coronal slices. Vaginal gel injection may, in some cases, improve visualization [2,8].

Management

In women with a completely septate uterus, a double cervix, and a longitudinal vaginal septum, without an adverse reproductive history, no surgical indication is currently recommended. However, resection of the vaginal septum may be considered in cases of dyspareunia [5].

In patients with a septate uterus and a history of infertility or miscarriages, hysteroscopic metroplasty may be proposed. This minimally invasive technique, particularly with the use of miniaturized devices, is associated with a low complication rate and favorable fertility outcomes, with improved pregnancy rates and reduced miscarriages according to several observational studies [1,3,5,6]. However, it should be noted that there is no strong statistical evidence supporting surgical indication in asymptomatic patients [5].

Techniques for uterine septum resection are not yet fully standardized, and the indication for surgery remains debated. The National Institute for Health and Care Excellence and the ASRM advocate for hysteroscopic septum resection, whereas the ESHRE and the Royal College of Obstetricians and Gynaecologists (RCOG) find the existing evidence insufficient to universally recommend this intervention [3].

Regarding other associated malformations, there is no surgical indication for a double cervix. However, the longitudinal vaginal septum may be resected in symptomatic patients, particularly in cases of dyspareunia, with several techniques described in the literature [6].

## Conclusions

Advances in pelvic imaging, particularly 3D ultrasound and MRI, have improved the diagnosis of complex congenital Müllerian anomalies, including the U2b C2 V1 anomaly, often avoiding the need for invasive procedures. The clinical impact of these malformations varies widely, ranging from asymptomatic cases to significant reproductive disorders. While management remains challenging due to the lack of standardized treatment protocols, some studies report improved fertility outcomes following hysteroscopic surgery. Therefore, a personalized therapeutic approach is recommended, especially in symptomatic patients, though further research is needed to establish best practices.
